# Predictive modelling of freeway space utilising clinical history, normalised muscle activity, dental occlusion, and mandibular movement analysis

**DOI:** 10.1038/s41598-024-67640-3

**Published:** 2024-07-16

**Authors:** Taseef Hasan Farook, Tashreque Mohammed Haq, Lameesa Ramees, James Dudley

**Affiliations:** Adelaide Dental School, The University of Adelaide, Adelaide, SA 5000 Australia

**Keywords:** Dentistry, Machine learning

## Abstract

This study aimed to predict dental freeway space by examining the clinical history, habits, occlusal parameters, mandibular hard tissue movement, soft tissue motion, muscle activity, and temporomandibular joint function of 66 participants. Data collection involved video-based facial landmark tracking, mandibular electrognathography, surface electromyography of mandibular range of motion, freeway space, chewing tasks, phonetic expressions, joint vibration analysis, and 3D jaw scans of occlusion. This resulted in a dataset of 121 predictor features, with freeway space as the target variable. Six models were trained on synthetic data ranging from 500 to 25,000 observations, with 65 original observations reserved for testing: Linear Regression, Random Forest, CatBoost Regressor, XGBoost Regressor, Multilayer Perceptron Neural Network (MPNN), and TabNet. Explainable AI indicated that key predictors of freeway space included phonetics, resting temporalis muscle activity, mandibular muscle activity during clenching, body weight, mandibular hard tissue lateral displacements, and dental arch parameters. CatBoost excelled with a test error of 0.65 mm using 5000 synthetic data points, while a refined MPNN achieved the best performance with 25,000 synthetic data points and 121 unique predictors, yielding an absolute error of 0.43 mm on the 65 original observations.

## Introduction

Interocclusal clearance, also known as dental freeway space, is the clearance between the opposing dental arches when the mandible is in its physiologic rest position^1^. It serves as a baseline for prosthetic restorations and influences occlusal relationships and vertical dimension in restorative dentistry^1^. Maintaining an adequate freeway space ensures stability, comfort, and function of prosthetic devices for the patient while preventing temporomandibular joint (TMJ) disorders and muscle fatigue. When anatomical variations are excluded, abnormal changes in freeway space can sometimes indicate active parafunctional habits such as bruxism^2^.

The equilibrium of freeway space is intricately tied to factors such as muscle tone, temporomandibular joint (TMJ) health, and dental occlusion^3^. Imbalances or tension in these components can affect mandibular resting position, simultaneously affecting the health of the TMJ complex. Issues like malocclusion, misalignment, tooth loss, prosthetic restorations, parafunctional habits, muscle fatigue, and aging contribute to changes in freeway space. Estimating freeway space in clinical dentistry is challenging due to its dynamic nature, influenced by various factors. Achieving precise measurements is complicated by individual variations, tooth loss, prosthetic restorations, and parafunctional habits, and the lack of standardized measurement techniques introduces subjectivity and inter-operator variability^2^.

Addressing these challenges requires a comprehensive understanding of the multifactorial influences on freeway space. Deep learning, a subset of machine learning, is a form of modern-day artificial intelligence-based predictive modelling, emerges as a promising approach to recognise patterns and variations in freeway space influenced by muscle tone, TMJ health, and dental occlusion. For example, convolutional neural networks (CNNs) can learn complex categorical relationships and potentially standardise the estimation process, reducing subjectivity and inter-operator variability, while regression-based models like XGBoost are able to predict continuous variables and can adapt to significant individual variations, accounting for factors like occlusal discrepancies and mandibular range of motion to provide a robust estimation of freeway space^4,5^.

### Study rationale

Despite potential advantages, the application of quantitative approaches to predict continuous variables, such as freeway space, through a multitude of predictor variables remains undocumented in peer-reviewed dental literature. Although deep learning models are increasingly deployed in dentistry, their perceived 'black box' nature has prompted researchers to explore methods for extracting explanations from these models^5,6^. The current study seeks to fill a gap in dental literature by pioneering the exploration of accurate freeway space prediction using diverse parameters processed through deep learning. Additionally, explainability models were used to decipher which parameters are prioritised by the deep learning models in rendering predictive decisions^6^. The parameters under consideration encompass clinical history, occlusal factors, mandibular movement evaluation, soft tissue movement analysis, normalised muscle activity derived from electromyography processed through deep learning, and non-invasive temporomandibular joint (TMJ) function analysis.

### Research aim

This current study aimed to predict dental freeway space by examining clinical history, occlusal parameters, mandibular movement, soft tissue motion, muscle activity from electromyography (EMG), and 3D intraoral scanning. It was hypothesised that accurate prediction of freeway space cannot solely rely on clinical history and non-invasive chairside investigative parameters.

## Materials and method

The University of Adelaide Human Research Ethics Committee (H-2022-185) granted approval for this study which also adhered to the Minimum Information for Clinical Artificial Intelligence Modelling (MI-CLAIM) 2021 protocol checklist^7^.

### Eligibility criteria

The eligibility criteria required participants to have most of their natural permanent teeth, with no more than one missing tooth per quadrant. If a first molar was missing, all other teeth in the arch had to be present. Individuals would be excluded if they had long-span edentulous arches, shortened dental arches, retained deciduous molars, or two or more fixed partial dentures or dental crowns. To promote randomisation and prevent accidental omission of individuals showing clinical signs of temporomandibular joint dysfunction but not self-reporting the condition, perceived symptoms or existing medical conditions were not used as exclusion criteria.

### Participant recruitment

**Figure 1:**
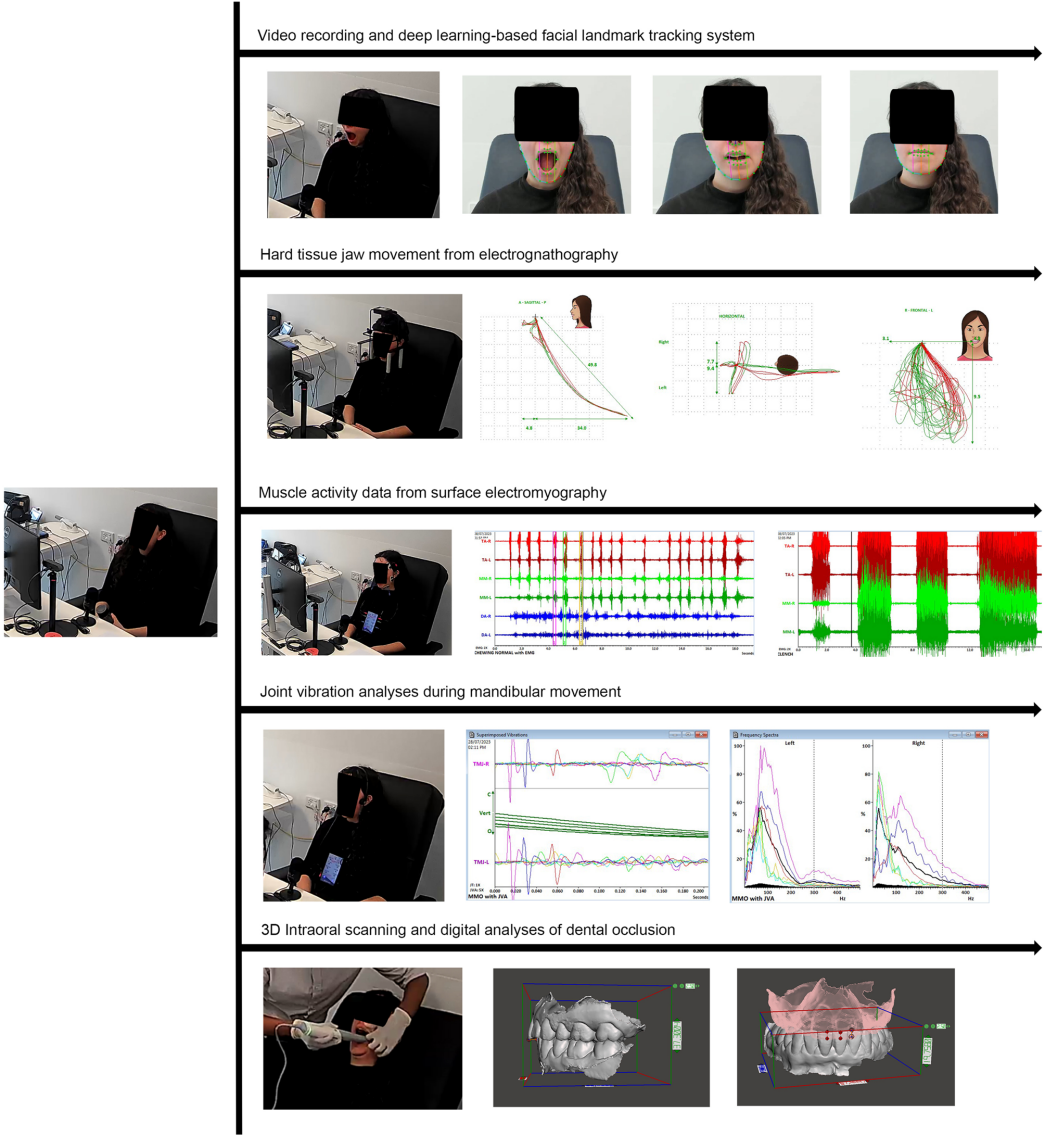
Clinical data collection workflow.

All experiments were performed in accordance with relevant guidelines and regulations. In mid-2023, 70 South Australian participants were recruited, with 66 completing the entire process after informed consent was obtained from all subjects/participants. EMG signals from four individuals were embedded with heavy noise generated by micro-electric conductions triggered by their facial vellus hair. Manual denoising would have substantially altered the original signal, so these four participants were excluded. Figure 1 demonstrates the steps of clinical data collection for the study and are explained in the subsections below. Prior to conducting the current research, systematic reviews were conducted on the most common sources of biases arising from human and device-dependent factors in jaw tracking and AI-based decisions concerning the temporomandibular joint complex^2,5,8,9^. The current research evaluations build upon a systematic series of prior investigations, documented in the following subsections.

### Clinical history taking

Participants initially completed a semi-structured questionnaire covering demographics, self-reported parafunctional habits, and temporomandibular dysfunction symptoms. Subsequently, they reported their orthodontic treatment history and medical conditions diagnosed by general medical practitioners.

### Facial landmark tracking

Participants were seated 45 cm from a consumer camera (Logitech Brio 4 K) and instructed to perform unassisted maximum mouth opening, maximum lateral excursion, and maximum anterior protrusion^10^. Each participant undertook a single session of video recording using the Brio-4 K camera at 1080p resolution and 60fps, utilising a 13-megapixel lens. The video outputs were at a native bitrate of 2500 Kbps, encoded using H.264 NVENC, and exported in Matroska Video (.mkv) format.

The video recordings were processed using a deep learning-based facial landmark tracking system to assess habitual head tilting patterns and soft tissue displacements during lateral excursions and speech, based on previous research implementations^11^. This was accomplished with a set of open-source in-house software developed by the authors, namely Dental Loop FLT^12^ v5.2 (https://github.com/ElsevierSoftwareX/SOFTX-D-23-00353) and Dental Loop SnP v1.0 (https://github.com/saadism777/Dental-Loop-SnP-Speech-and-Phonetic-Pattern-Recognition).^13^ The software performed facial landmark detection and tracking for both retrospective data and real-time tracing, utilising the OpenCV and Dlib packages coded to PEP-8 standards^12,13^. It introduced custom soft-tissue cephalometric landmarks for continuous measurements and displayed video statistics in a resizable OpenCV window. The outcomes were stored and automatically tabulated, thereby avoiding errors commonly associated with operator-based image tracking and segmentation.

### Digital electrognathography

Participants were then instructed to repeat the same activities attached to an electrognathograph (EGN) (JT-3D; BioResearch Associates Inc). Each activity was repeated three times, and mean values across all displacements on the vertical, lateral, and sagittal planes were quantified in millimetres using the manufacturer-provided software suite (BioPak v8.9; BioResearch Associates Inc). Freeway space was measured by instructing the participants to assume a rest vertical position and calibrating the electrognathograph to read this position as 0 displacement. Participants were then asked to bring their mandibles to the occlusal vertical dimension and tap their teeth together twice. The vertical displacement values were recorded for both taps. This procedure was performed according to the manufacturer's recommendations. The process was repeated three times per participant, and the average displacement was recorded in millimetres.

Subsequently, participants engaged in chewing soft sugar-free gum, adhering to specified durations of 15 s on each side and an additional 20 s where they chewed the gum naturally. Quantitative values for vertical and slanted range of motion were recorded. Participants were then prompted to perform phonetic expressions of specific consonants (fricatives, sibilants, linguodental, and bilabial) while the EGN device remained attached. This was followed by asking the participants to pronounce the numbers from 61 to 69, which provided an insight of the variations in jaw movement while transitioning across the four specific consonants. The mean mandibular displacement during the pronunciation of each consonant was recorded. The speech scripts were derived from established English sentences outlined by Cheireici et al. in 1979^14^.

### Surface electromyography

Participants were linked to an electromyography (EMG) unit (BioEMGIII; BioResearch Associates Inc) and instructed to replicate the activities recorded with the 6-channel surface electrognathography (EGN) unit with TENS-based grounding. The sampling rates for mouth opening, lateral excursions, and anterior protrusion were 3000 Hz. Additionally, participants performed unstimulated clenching, which involved bringing their teeth together and clenching without any external object affecting occlusal contact. They then demonstrated their maximum bite force on rolls of cotton positioned bilaterally in the molar regions. Sampling rates for chewing, clench, and maximum bite force were set at 1000 Hz. The amplifier gains for all channels were retained at the default 5000.

Standardisation across all 66 participants was achieved by normalising signal sweeps for each activity using an in-house deep learning-based software, which produced standardised quotients for both muscle intensity and activity duration^15^. The EMG images were standardised to range between 1604 × 579 pixels and 1617 × 590 pixels, with padded normalisation to ensure that the resulting signal had a standard length of max(M, 5)− min(M, 5) + 1^16^. These normalisation methods were implemented following previous evaluations^16^, that were subsequently repurposed to create an in-house, open-source signal processing tool, Dental Loop Signals v1.0 (https://github.com/SoftwareImpacts/SIMPAC-2023-498)^15^.

Dental Loop Signals offers functions such as image display, muscle selection, signal extraction, and clustering. The NeuroKit2’s EMG processing API was used to calculate intensity and duration quotients, while the Cluster class handled clustering algorithms based on muscle activity^17^. The software was made modular to support adjustable parameters, activity specification, batch processing, muscle selection, and custom labelling.

### Joint vibration analyses

Temporomandibular joint function activity was recorded using a non-invasive vibration analysis tool (JVA; BioResearch Associates Inc), capturing variations in vibration integral, amplitude, and frequency for both left and right joints. The data was processed alongside EMG data following previously published methodologies^18^. Sampling rate was set at 5000 Hz and frequency range optimisation for vibrations within 0.5 Hz to 300 Hz.

### 3D intraoral scans

Finally, an intraoral scanner (Shining3D AoralScan 3) was utilised to scan upper and lower arches with occlusion following previously established scanning protocols for optimum detailed impressions^19^. A pilot evaluation assessed the hardware used and operator-induced biases, revealing that neither the choice of hardware nor the clinical experience of the operator influenced machine predictions^20^. It was also found that mobile devices were capable of capturing data comparable to standard workstations^21^ meaning medical devices and graphics processing units connected through highspeed terminals such as USB-4 and Oculink are capable of generating accurate sampling data without signal loss. CAD software (Meshmixer; Autodesk Inc.) measured inter-canine and intermolar distances, arch perimeters, overjet, and overbite.

### Data compilation for post-processing

The combined data sheet obtained in post-processing included a comprehensive set of features related to occlusal parameters such as inter-canine and inter-molar relationships, arch perimeter, overbite, overjet, and various ranges of motion. It also covered habitual head tilt, soft tissue lateral excursion, chewing patterns, EGN data of range of motion and mean freeway space, and normalised EMG data of muscle intensity and activity duration, joint vibration analysis metrics, and phonetic expressions. Additionally, personal, and health-related information, including ethnicity, age, gender, height, weight, BMI, dental history, medical conditions, and lifestyle factors, such as jaw clenching, bruxism, sleep apnoea, and diagnosed health conditions were gathered. These were all combined to form a dataset of 121 predictor features and one continuous target variable (Freeway space). One observation was excluded following data compilation owing to a mismatch in categorisation.

### Predictive modelling and explainability

Sixty-five original observations were finally available. Synthetic observations were generated using the Synthetic Data Vault (SDV) Python library using a tuned Triplet-based Variational Autoencoders (TVAE)^22^.

To model the data, six different approaches were considered: Linear Regression, CatBoost, XGBoost, Random Forest, Multilayer Perceptron Neural Networks (MPNN), and TabNet TabNet^4,23,24^. Each model offered unique approaches to handle the variables. Traditional Linear Regression makes predictions based on the assumption of linear relationships. CatBoost handles categorical data through iterative learning, or boosting. XGBoost uses branching decision trees instead of strictly linear relationships, while Random Forest combines predictions from multiple decision trees. MPNNs evaluate information in sequential layers and are structured to handle complex relationships. TabNet uses an attention mechanism that focuses on combinations of features in tabular data that the model deems relevant and of higher priority in establishing relationships. Linear Regression was used as the baseline model. Hyperparameter tuning was conducted with fivefold cross-validation for XGBoost, CatBoost, and Random Forest, while MPNN and TabNet were validated using an 80:20 synthetic data split for training and validation^25,26^.

**Figure 2:**
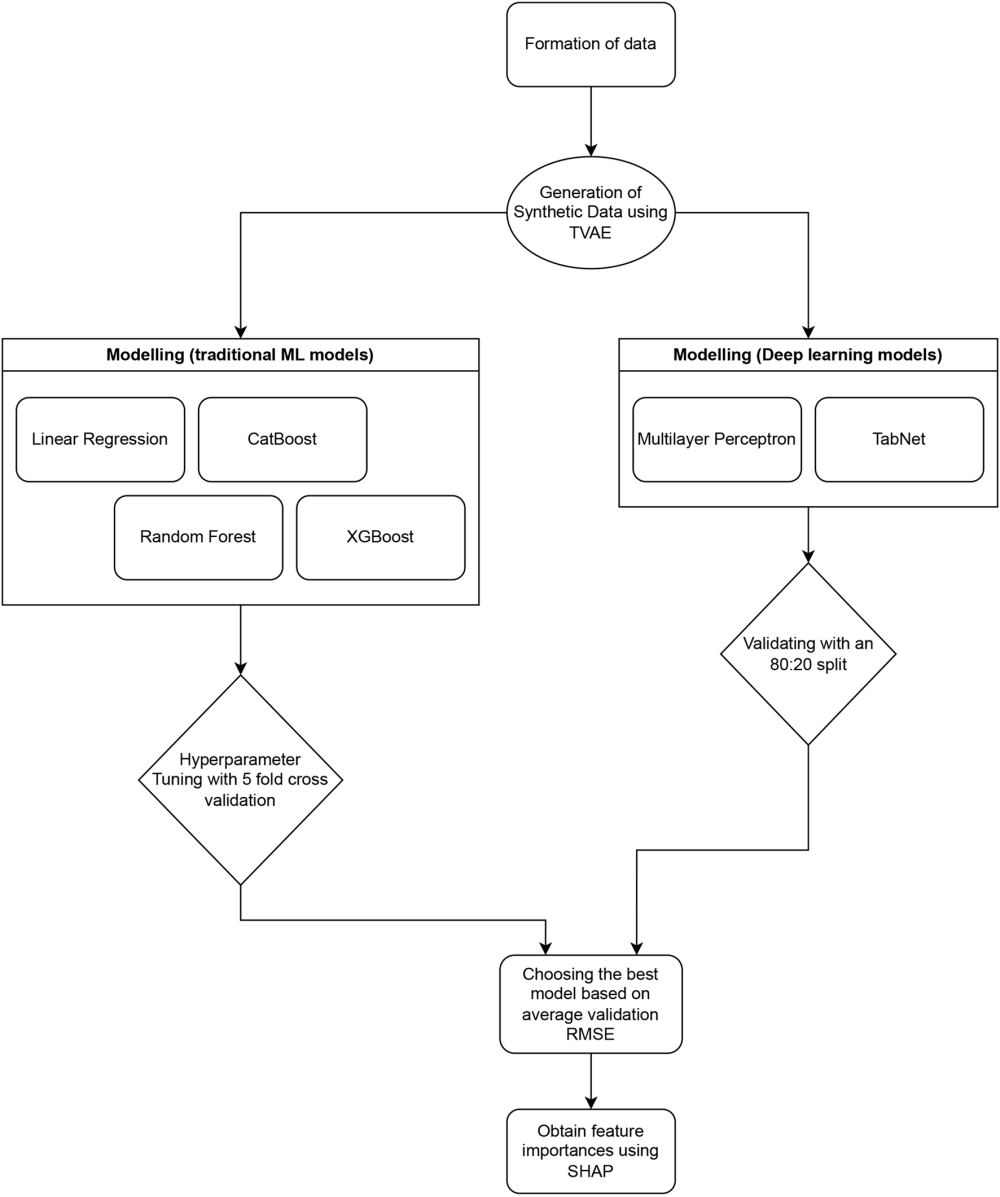
Predictive modelling workflow.

The best models were chosen after hyperparameter tuning to minimise the average validation root mean squared error (RMSE). Mean absolute error (MAE) in the current study served to quantify the average prediction accuracy by measuring the magnitude of errors between predicted and actual values, effectively handling biases introduced by outliers. Box plots were used to assess the distributional impact of categorical variables, while scatterplots and correlation analyses were utilized to evaluate the suitability of numerical variables. The overall workflow for predictive modelling is outlined in Fig. 2.

### Explainable AI

SHapley Additive ExPlanations (SHAP) was finally used to generate an explainability report of the importance of the 121 features. This is based on the Shapley values, developed from fair credit allocation in cooperative game theory which in the current instance computed values for features that produced the greatest contributions in predicting freeway space^27^. Based on the feature importances, the synthetic training data size was adjusted from 500 to 25,000 data until the best performing models were identified and tuned. Access to the codes, hyperparameter tuning information and SHAP analyses outcomes have been presented as supplementary information within the Data Availability subsection.

### Ethical approval

The study was approved by the University of Adelaide Human Research and Ethics Committee (HREC H-2022-185). All experiments were performed in accordance with relevant guidelines and regulations. Informed consent was obtained from all subjects for both study participation and dissemination of data through peer-reviewed publications.

### Consent of the participants

All participants provided written and signed consent prior to participating in the research.

## Results

The assessment of explainability revealed the most effective deep learning models prioritised sequential recitation from sixty-one to sixty-nine (60 s phonetics), temporalis muscle clenching EMG signals, temporalis muscle intensity during lateral excursions, body weight, and mandibular displacements during fricative phonetic expression as pivotal variables for accurate freeway space prediction. Subsequently, an in-depth analysis focused on the individual significance of features in the best-performing model, specifically for self-reported history. Aside from age, height, and body weight, elements like habitual head tilting and a positive history of clicking emerged as noteworthy factors influencing the model's decision-making process. After examining electrognathographic data, it was observed that phonetic expression, chewing patterns, and vertical displacement of the mandible during maximum mouth opening surfaced as the primary predictor features. All aspects of muscle functions and vibration analyses were deemed important, with temporalis muscle activities during clenching and joint vibration parameters during lateral excursions standing out as the most influential predictors for the optimal model in forecasting freeway space. Lastly, in the context of predictors derived from 3D models of the jaws, maxillary intermolar distance exhibited slightly higher importance across the board. The list of predictors and their associated SHAP values have been listed in Table 1**.**

**Table 1:** Included predictors and their associated SHAP values in descending order.

	**SHAP values**
**History reporting and video-based image tracking**	
Weight (kg)	0.042053616
Height (cm)	0.020327741
Age	0.017703816
History of recurrent sinus infections	0.016151707
Habitual Head tilt while sitting	0.01593526
History of jaw clicking	0.013174334
Body mass index	0.011792403
Prescribed with long term medication	0.010807923
Positive history of jaw lockout in last 2 years	0.010507693
Self-reported habit of bruxism	0.00986969
Positive history of one or more 3rd molar extractions	0.009736395
Medically diagnosed Asthma	0.008468335
Positive history of orthodontic treatment	0.00659521
persistent nasal congestion	0.006431958
Positive history of jaw pain in the last 2 years	0.006141438
persistently chapped lips	0.005522408
Self-reorted history of sleep apnoea	0.004899097
Biological male	0.004792741
Positive history of lisp	0.004662612
Medically treated for mental health condition	0.004583376
Persistent dry mouth	0.004177276
Self-reported habit of jaw clenching	0.004045596
self-reported habit of stuttering	0.003946807
Positive history of endocrine disorder	0.00364065
Asian Ethnicity	0.003559383
Positive history of Adenoid or Sinus surgery	0.003473617
self-reported habit of nail or object biting	0.003377221
Caucasian Ethnicity	0.003219294
Positive history of one or more Inflammatory disorders	0.003041349
Medically diagnosed Vitamin D deficiency	0.002696542
Endodontic and restorative work done in the last 2 years	0.002439181
Indian Ethnicity	0.002310901
Persistently increased thirst	0.002308223
Positive history of dysphagia	0.002201416
Positive history of hypertension or high cholesterol	0.002113167
Positive history of jaw injury	0.00200944
Biological female	0.001900502
**Hard tissue jaw movement from electrognathography**	
"60 s" phonetics vertical	0.052871694
Fricative vertical	0.03978942
chewing right side lateral right	0.034802932
Linguodental phonetics vertical	0.031100669
Range of motion lateral left	0.030884674
Range of motion slant	0.028871148
chewing right side vertical	0.027770698
Range of motion lateral right	0.027029142
"60 s" phonetics Lateral left	0.026270432
Fricative Lateral left	0.025380253
Range of Motion MAP	0.025355571
chewing left side vertical	0.023793156
Bilabial phonetics lateral left	0.023682554
Sibilant lateral right	0.023248551
Range of motion vertical	0.022846649
chewing normal side lateral right	0.02258304
Linguodental phonetics lateral left	0.021346865
chewing normal side vertical	0.021191025
Bilabial phonetics vertical	0.018299019
chewing left side Lateral left	0.018252344
Sibilant vertical	0.017686127
chewing right side Lateral left	0.017319617
chewing normal side Lateral left	0.016566678
Linguodental phonetics Lateral right	0.016271831
Fricative protrusion	0.015298883
Fricative Lateral right	0.015003637
chewing left side Lateral right	0.014516858
Linguodental phonetics protrusion	0.014204259
Sibilant protrusion	0.014107713
"60 s" phonetics protrusion	0.013451073
Bilabial phonetics Lateral right	0.013187866
"60 s" phonetics Lateral right	0.012768034
Bilabial phonetics protrusion	0.011844572
Sibilant Lateral left	0.011623253
chewing left side protrusion	0.011365345
chewing normal protrusion	0.010647815
chewing right side protrusion	0.008172464
**Muscle activity data from surface electromyography**	
CLENCH TA-L	0.046401279
MMO duration MM-L	0.03429114
MAP duration TA-R	0.026843144
MMO duration DA-R	0.025342023
MAP intensity TA-L	0.024132729
MAP intensity TA-R	0.022744113
Maximum bite force TA-L	0.019701407
MLE duration TA-R	0.018560376
MAP intensity DA-L	0.016358545
MLE duration MM-L	0.01583352
MMO duration MM-R	0.015562302
MMO intensity MM-R	0.01541303
MAP intensity MM-R	0.014816482
MLE intensity TA-R	0.013161449
MAP duration MM-L	0.01248169
Maximum bite force MM-L	0.011613198
Maximum bite force MM-R	0.011368616
MLE intensity DA-R	0.01065957
MMO duration TA-R	0.009915707
MLE duration DA-R	0.008340101
MAP duration DA-L	0.007337743
**Joint vibration analyses during mandibular movement**	
JVA MLE right Integral ratio	0.035678653
JVA MAP left integral	0.028614507
JVA MAP left Integral ratio	0.026966083
JVA MLE left median frequency	0.020164756
JVA MAP right integral > 300 Hz	0.018249705
JVA MMO left integral ratio	0.01724231
JVA MMO left peak frequency	0.016088684
JVA MLE left integral ratio	0.016008068
JVA MMO right median frequency	0.014769565
JVA MLE right integral < 300 Hz	0.013615672
JVA MAP left integral < 300 Hz	0.013207504
JVA MAP right integral < 300 Hz	0.012381763
JVA MAP left peak frequency	0.01179058
JVA MLE left integral < 300 Hz	0.011390415
JVA MLE left peak amplitude	0.010760952
JVA MMO right integral > 300 Hz	0.009839767
JVA MAP right integral	0.00979051
JVA MLE left integral	0.009492189
JVA MMO left integral < 300 Hz	0.007800211
JVA MMO right integral	0.007071045
JVA MMO left integral > 300 Hz	0.005547697
**3D intraoral scanning and digital analyses of dental occlusion**	
Inter-molar distance maxilla	0.035511825
Arch perimeter maxilla	0.028746161
Overbite	0.028572159
Inter-molar distance mandible	0.026525288
Inter-canine distance maxilla	0.022977784
Overjet	0.020070627
Arch perimeter mandible	0.017770107
Inter-canine distance mandible	0.012589872

*MMO* Maximum mouth opening, *MAP* Maximum anterior protrusion, *MLE* Maximum lateral excursion, *TA-L* Left Temporalis, *TA-R* Right Temporalis, *MM-L* Left Masseter, *MM-R* Right Masseter, *DA-L* Left Digastric, *DA-R* Right Digastric, *JVA* Joint Vibration Analysis, *MMO* Maximum mouth opening, *MAP* Maximum anterior protrusion, *MLE* Maximum lateral excursion.

Without exclusion of any low-ranking predictors, the resulting models were first evaluated on the test set of 66 original observations. Notably, CatBoost outperformed other models with a test MAE of 0.65. Multilayer Perceptron achieved the best performance with a test MAE of 0.556, surpassing TabNet, which demonstrated signs of overfitting. Once low-ranking features were eliminated, a synthetic dataset of 5000 data was generated to train each model and then test it on the original datasets. The boosted tree base model, CatBoost, performed best, with a final test MAE of 0.69. Attempting further improvement, features with importance scores ≥ 0.5 were retained, yet the final test MAE using CatBoost remained at 0.70.

It was assumed that models exclusively trained and validated on synthetic data lacked exposure to variables present within real observations. To investigate this theory, the subsequent evaluation excluded no low-ranking features, but the training dataset was dropped to 500 synthetic observations generated using TVAE. Columns with shape similarity scores of 0.75 and above were retained. Subsequently, 30 randomly selected observations from the original data were randomly incorporated into the synthetic dataset, while the remaining 35 were left unseen for testing. This process aimed to challenge the initial theory. The entire modelling process was repeated, and subsequent scores were obtained for evaluation (Table 2).

**Table 2:** Errors (in mm) in estimating freeway space for different deep learning models after training on 500 synthetic data.

Models	Validation RMSE	Test RMSE	Test MAE^c^
Linear regression^a^	0.6174	1.1659	0.8624
Random forest^a^	0.6028	1.0369	0.7565
Catboost regressor^a^	0.5965	1.0915	0.8027
XGBoost regressor^a^	0.6344	1.1772	0.8617
Multilayer perceptron neural network^b^	0.5264	0.9970	0.7619
TabNet^b^	2.2482	12.4478	4.3147

^a^validation RMSE performed by fivefold cross validation of synthetic data.

^b^validation RMSE performed after 80:20 split of synthetic data.

^c^Test RMSE and MAE on 65 original observations from human participants.

Following unsatisfactory outcomes from testing the previous theory, a substantial 25,000 synthetic observations were generated using TVAE. Only columns with shape similarity scores of 0.6 and above were retained. Unlike the previous scenario and similar to the first scenario, the original data remained untouched, with no observations transferred to the synthetic dataset. The use of a large number of observations aimed to prevent underfitting. The modelling steps were then repeated, and the ensuing results were recorded (Table 3). Analysing the results, the CatBoost boosted tree model exhibited an improved MAE score of 0.65 comparable to the previous iteration. The Multilayer Perceptron Neural Network (MPNN) outperformed the more complex models, achieving a test MAE of 0.556. In contrast, the more intricate Tabnet model demonstrated poorer generalisation, indicating potential overfitting. The Multilayer Perceptron hyperparameters were adjusted further to produce featured hidden layer sizes of 128, 512, 256, and 64, a 25% dropout layer, ReLU activation function, Adam optimizer, early stopping with a patience of 50, a validation split of 25%, and 1000 epochs, yielding a significantly reduced test mean absolute error of 0.4258 mm.

**Table 3:** Errors (in mm) in estimating freeway space for different deep learning models after training on 25,000 synthetic data.

Models	Validation RMSE	Test RMSE	Test MAE^c^
Linear regression^a^	0.64150	0.90344	0.70632
Random forest^a^	0.61012	1.13127	0.91469
Catboost regressor^a^	0.54958	0.87152	0.65129
XGBoost regressor^a^	0.65363	1.05454	0.80435
Multilayer perceptron neural network^b^	0.53229	0.725154	0.55635
TabNet^b^	0.56171	0.909903	0.71141

^a^validation RMSE performed by fivefold cross validation of synthetic data.

^b^validation RMSE performed after 80:20 split of synthetic data.

^c^Test RMSE and MAE on 65 original observations from human participants.

## Discussion

The primary objective of this study was to construct a prediction model for freeway space based on a comprehensive assessment of jaw movement and self-reported history in a cohort of 66 individuals in South Australia. The precision of the freeway space predictions across all 66 individuals was determined to be 0.43 mm, surpassing the documented human error in maxillofacial index measurements, which is approximately 1.0 mm^28^, leading to the rejection of the initial hypothesis.

Freeway space in dental practice demands a measurement precision typically within the range of 0.2 to 1.0 mm^29^. Variations stem from factors such as muscle relaxation affecting mandibular position, variations in head posture during measurement, and differences in technique or instrument calibration. Although calibration tools aim to mitigate these variations, natural fluctuations remain unavoidable and cannot be precisely quantified without considering all variables of dynamic jaw movement collectively. The top-performing model in this study achieved an error rate of 0.43 mm, surpassing the human-derived average standard variation of 0.5 mm across different devices.

Notably, this study stands out as the first to extract explanations from deep learning models in estimating dental freeway space, shedding light on the influential features guiding their decisions. Medical insights further elucidate the rationale behind the models' ranking choices. Age-related alterations in muscle tone and joint structure, often imperceptible to human observation, influence the resting position of the jaw and impact freeway space. Height and weight variations contribute to the complexities of craniofacial morphology, potentially influencing spatial relationships, including freeway space. Soft tissue lateral excursion, indicative of movement during lateral jaw movements, significantly influences resting position and freeway space when imbalances or restrictions are present, subsequently affecting hard tissue lateral excursion. Habitual head tilting induces changes in the mandible's position, thereby altering freeway space and influencing occlusal relationships, muscle activity, and bite stability^30^.

Subtle imbalances in arch perimeter, overjet, or overbite directly impact spatial dimensions, potentially leading to insufficient freeway space, discomfort, instability, or functional challenges. Prediction models aim to discern unique trends in individuals, capturing minute variations in growth, speech patterns, and mandibular lateral excursions. Clear articulation during speech expression necessitates adequate freeway space. Insufficient freeway space, whether due to malocclusion or pathophysiological variations in the TMJ complex, may affect patients' phonetic patterns, resulting in articulatory challenges during mandibular translation^31,32^. These variations, often imperceptible to the naked eye but detectable through optical landmark tracking and EGN, provide the models with sufficient data diversity to establish trends. Intriguingly, prediction models and deep learning excel in identifying minor deviations from common trends, whereas human observation tends to focus on similarities and familiarity.

Constricted mandibular range of motion can compromise freeway space, limiting the resting position and impacting occlusal stability and patient comfort. In cases of inadequate bimaxillary clearance, unstable unilateral chewing dynamics may result^33^. Explainability analyses indicated that AI performed better when considering phonetic expression, clenching muscle activity, and TMJ vibrations during lateral and anterior protrusion. This preference is likely because the models excel at learning from nuanced variations in human behaviour rather than generic movements that may be consistent across individuals to some extent. Additionally, the models identified maxillary intermolar distance from 3D models as a significant predictor. This finding is intriguing, considering a previous study reporting the long-term effects of maxillary expansion, which observed the greatest changes and highest relapses in intermolar regions, deeming inter-canine distances less reliable measures^34^. In the current study, inter-canine distances were ranked at the bottom of the priority list by the models.

Persistent clenching and bruxism, leading to occlusal changes, tooth wear, and muscle tension, have an important influence on the vertical dimension at rest^33^. Some of these parafunctional habits are more prevalent in younger patients and often necessitate orthodontic intervention^35^. Orthodontic treatment addressing misalignment resulting from parafunctional habits or malocclusion derived from arch space discrepancies can inadvertently alter freeway space^36^. The top-performing models considered all these factors in their predictions without requiring operators to rank features for the deep learning models. Variations in maximum occlusal force can impact tooth articulation and muscle tension within the freeway space^37^. However, the subjective nature of maximum occlusal force and its variability within individuals have been subjects of debate in dentistry; interestingly, the models in this study ranked these parameters lower on their priority list^38,39^.

While self-reported history is susceptible to inaccuracies, clinicians often prioritise quantifiable clinical evidence over patient-reported conditions due to potential biases^40^. However, the exclusion of self-reported history in the initial design of the current study, relying solely on numerical variables, led to some absolute errors. This underscores the role of self-reported history in predicting functional freeway space. Even when imaging data is available, self-reported history serves as a secondary data source for assessing the overall health of the temporomandibular joint^5,8^. The study suggests that while diagnosing the joint complex may not heavily depend on history-taking alone, evaluating occlusal parameters, especially freeway space, necessitates a comprehensive history for accurate function prediction.

The present study faced several limitations, including a shortage of original observations, prompting the synthesis of 25,000 data points for model training. Despite efforts, generating 5,000 synthetic data over 500 did not yield substantial improvements. The study highlighted the potential for enhanced accuracy with a larger original dataset, as evidenced by the neural network model achieving a 0.43 mm error on the original unseen dataset of 65 participants. While precision below 0.1 mm is optimistic with a larger dataset with consolidated features, the current report did not explore the consolidation of features from text and images into a unified multimodal AI^41^ an approach that warrants further research. Furthermore, excluding true edentulous participants in the current study might present a significant challenge in determining freeway space for such patients in clinical implementation. Many of the predictive features identified in the results may not be applicable in edentulous cases. Future research could investigate how the model predictions would perform if features that do not exist or are significantly impacted by edentulism were excluded from the input.

Nevertheless, the study emphasised the efficacy of synthetically generated data in training neural network models with minimal error, showcasing their capability to predict freeway space using medically relevant yet highly variable data. The results hold promise for the implementation of decision support tools for clinicians, utilising computerised models to assess clinically relevant parameters such as expected freeway space based on an individual patient's unique set of clinical attributes.

## Conclusion

The evaluation of deep learning models highlighted specific variables, including phonetic expression, temporalis muscle activity, mandibular muscle activation during clenching, and mandibular lateral range of motion as potentially important parameters that infuence freeway space prediction. Notably, self-reported history of posture altering habits such as head tilting also played a noteworthy role. The comprehensive analysis further emphasised the prominence of predictive modelling that demonstrated a commendable precision of 0.43 mm in predicting freeway space from 121 unique jaw movement parameters.

## Data Availability

All supplementary information and codes to the models have been provided in a Github repository: https://github.com/Tashreque/predictive-modelling-dental-freeway-space (last accessed 14 June 2024).
